# Mostafa Maged sling technique to treat prolapsed uterus in an easy way (New Technique)

**DOI:** 10.4314/ejhs.v33i5.22

**Published:** 2023-09

**Authors:** Mostafa Maged Ali

**Affiliations:** 1 Obstetric and Gynecological Department, Fayoum General Hospital, Fayoum, Egypt

**Keywords:** Mostafa Maged, prolapse, uterus, heaviness, sling

## Abstract

**Background:**

In the female population, pelvic organ prolapse is a common problem that lowers people's quality of life in terms of their health. Depending on the severity of the prolapse and the symptoms, there are many treatment options. Simple observation, vaginal pessaries, or surgical management are all possible treatments. Reconstructive pelvic surgery with or without mesh augmentation and obliterative surgery are two surgical treatments that are available. Due to the contentious concerns surrounding the use of mesh and the rising demand for uterine preservation, surgical practices are currently shifting.

**Methods:**

Just two cases are included in this study due to the rarity of this condition. In this study, I introduce a new technique to the literature (Mostafa Maged sling technique) which will be challenging. This technique depends on round ligaments and ovarian ligament to hitch up the whole uterus.

**Results:**

There were no difficulties following the procedure. None of the patients required blood transfusions, and there were no signs of dehiscence or incision infection. Non-steroidal antiinflammatory medications were administered to both patients as analgesia. On the first postoperative day, the foley catheters were removed from both patients.

**Conclusion:**

A simple and new manueuver is applied in the literature to treat the uterine prolapse. It is easy to learn and easy to perform. We need further studies to compare different techniques including Mostafa Maged sling operation to manage uterine prolapse.

## Introduction

The failure of the ligamentous and fascial supports causes the uterus to herniate into or beyond the vagina, which is known as uterine prolapse (1).

Prolapse risk factors include older age, race, family history, higher parity, increased body mass index, and increased body fat. Constipation, vaginal birth, intrapartum conditions (macrosomia, prolonged second stage of labour, episiotomy, and epidural anesthesia), increased abdominal pressure, and menopause are some of these (1).

Pelvic organ prolapse is a general term that refers to a variety of diseases, including uterine prolapse. The best course of action to treat uterine prolapse depends on the precise defects that are present and factors like the patient's age, comorbidities, level of activity, desire for future fertility, history of prior prolapse surgery in other compartments, and patient preference. It also relies on the surgeon's skill and comfort level with the specific surgery. (2)

The ovarian ligaments, which are attached to the posterolateral side of the uterus, and the round ligament, which aids in keeping the uterus in an anteverted position during pregnancy, are the two ligaments that were used in this study. It is a strong and flexible structure, the round ligament. The cardinal ligaments support the uterus. (3) The most popular technique is vaginal hysterectomy with suspension of the vaginal cuff, which is currently the most common treatment for individuals with symptomatic uterine prolapse. However, uterus-preserving methods are becoming more and more well-liked. There is still no agreement on the best operation method as of right now. Despite the conflicting evidence in the literature, it has been hypothesized that hysterectomy may harm the pelvic floor's nerve supply and support structures. Therefore, after a vaginal hysterectomy, women may be more susceptible to bladder dysfunction and newly developing stress incontinence. (4,6)

Sacrospinous fixing has been proven to be a safe and effective treatment for uterine or vaginal vault prolapse in numerous retrospective and prospective trials. The sacrospinous ligament and the cervix are connected by two sutures, raising the apex above the levator plate. (5) Conservative therapies include mechanical (insertion of vaginal pessaries) and physical (pelvic floor muscle training) procedures to support the prolapse and enhance the function and support of the pelvic floor muscles, respectively. They are frequently provided for prolapses with lower degrees and to women who are unable or reluctant to undergo surgery. (7)

## Methods

All relevant information, like the purpose and methodology of the experiment, was explained to study participants beforehand, and informed consents was obtained from patients to be included. This case report study was conducted on two patients. All participants were subjected to a detailed history and a general examination to exclude the presence of any disorders as hypertension, diabetes, collagen diseases, kidney or liver diseases and hematological diseases; gynecological examinations were performed. The inclusion criteria are women who have uterine prolapse that affects their quality of life although they tried the medical treatment and kegel excercises , no abnormal findings other than pelvic organ prolapse on gynaecological and ultrasound examination (sonographically normal uterus, and ovaries), women who do not want to get pregnant or have reached the end of their fertility, and women who choose the new procedure and its potential drawbacks to the traditional methods.

The exclusion criteria are any present or past Gynecological tumours, uterine leiomyomas, endometriosis, past endometriosis surgery, and past incontinence or pelvic organ prolapse surgery. Grading of pelvic organ prolapse was done in the lithotomy posture with a full bladder.

**Case 1**: A female patient aged 49 years old who had delivered six children by vaginal route presented with heaviness at the pelvic area and a uterus protruding to the hymen diagnosed as (grade 3 uterine prolapse). She did not receive any treatment for any diseases and her BMI was 28 kg/m2.

**Case 2**: A female patient aged 56 years old who had delivered four children by vaginal route presented with heaviness at the pelvic area and (grade 2 uterine prolapse) was diagnosed. She was hypertensive and her BMI was 27 kg/m2.

## Mostafa Maged Sling Technique

**Figure 1:**
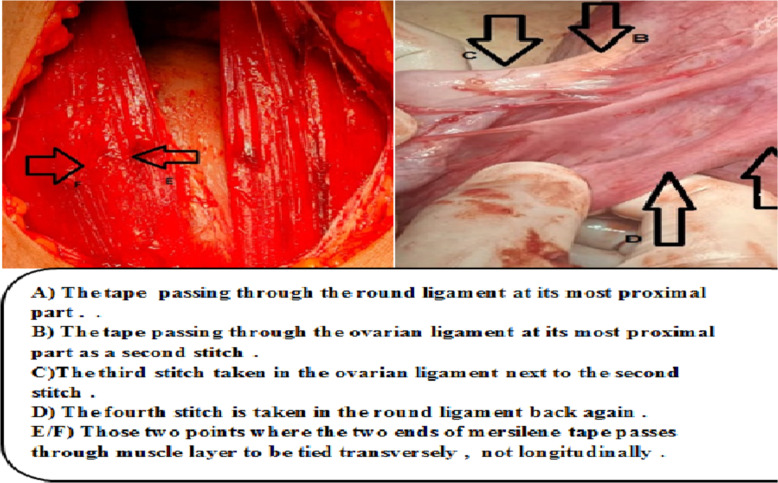
Steps of Mostafa Maged sling technique

**Figure 2:**
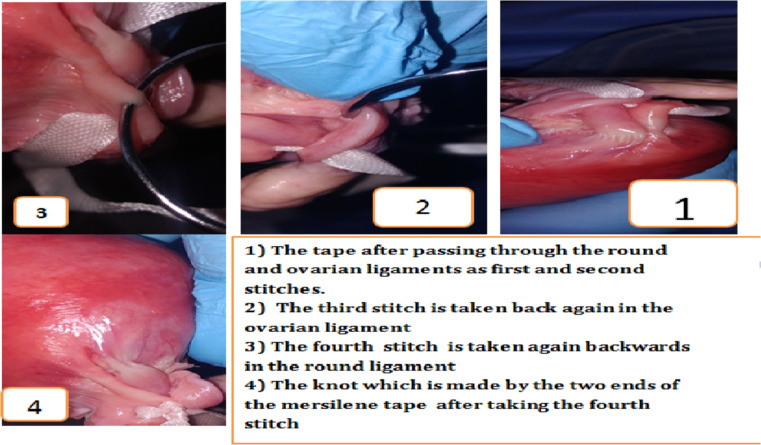
Steps of Mostafa Maged sling technique

This technique is well illustrated in Figure 1 and Figure 2.

Spinal anesthesia was taken for both patients. We make Pfannenstiel incision till we get to the intraperitoneal space. Also, while the uterus was repositioned upwards through the vagina, I palpated the uterine fundus. After grasping the uterus by My hands at the level of the incisional line at which we want to sling the uterus. We take these steps using the non-absorbable mersiline tape:

First, I take a suture in the round ligament in the most proximal part of the ligament (proximal to the uterine body). Second, I pass the needle to reach the utero-ovarian ligament to get this ligament attached directly to the round ligament.

Third, I take a stitch in the utero- ovarian ligament in the most proximal part of the ligament (proximal to the uterine body). Then, I take another stitch in the same ovarian ligament next to the first one distally. Fourth, I go back to the round ligament piercing it next to the previous stitch (mentioned in the first step). Then, we tie the thread to the first knot taken in the first step. Fifth, I pass the mersilene tape through the peritoneum and muscle layer of the anterior abdominal wall. Next, I make a knot at this level on the muscle layer of anterior abdominal wall. Sixth, we pass both needles through the sheath, and then, I make another knot at this level of sheath as well, just to support the uterus with three knots. Seventh, I make the same steps on the other side as well.

By this method, we increase the strength of the sling and avoid loosing of Mostafa Maged technique by suturing the two ligaments together and by taking three knots at different three levels (first level at ligaments, second level at anterioe abdominal muscle layer, third level at sheath). Then, I close the anterior abdominal wall. Eighth, I make the anterior and posterior vaginal walls repair for both patients (anterior and posterior colporrhaphy).

Optimal surgery was defined as uterine descent less than or equal to grade 1. Patients were checked in lithotomy position during follow-up visits for both the healing of the lower genital tract deficiency and a potential recurrence of POP using a bimanual digital exam while being instructed to do the valsalva maneuver. Also, ultrasonography was carried out to visualize where the uterus was.

## Results

There were no difficulties following the procedure. None of the patients required blood transfusions, and there were no signs of dehiscence or incision infection. Nonsteroidal anti-inflammatory medications were administered to all patients as analgesia. On the first postoperative day, the foley catheters were removed from both patients. Follow-up is made one month after the operation for both patients. Bi-manual examination was done and the uterus of both patients were still less than grade 1.

## Discussion

Despite the availability of conservative treatments, women who undergo surgery for pelvic organ prolapse run a 10-to-20% lifetime risk. The quality of life for women with pelvic organ prolapse can be improved with surgical procedures, according to a new systematic review and meta-analysis of RCTs (7,8). Education level and the conviction that the uterus contributes to one's sense of self were predictors of preference for uterine preservation, while the doctor's judgement, the likelihood of surgical complications, and the likelihood of malignancy were the most crucial considerations when deciding whether or not to proceed with surgery. There are several uterine-preserving treatments for apical prolapse that have been documented, but there are not many prospective RCTs that compare them to vaginal hysterectomy (8).

According to this theory, hysterectomy for uterine prolapse may be followed by a recurrence of pelvic organ prolapse, which turns out to be a serious problem. Meshes are frequently employed in the treatment of pelvic organ prolapse recurrence to reduce the recurrence. However, mesh complications are a major source of worry. Also, due to the harm done to the pelvic nerves and other tissues that support the pelvis following hysterectomy, incidence of urinary incontinence increases.

The benefit of avoiding difficulties unique to hysterectomy should be weighed against the possibility of developing uterine abnormalities later on and the unknown nature of upcoming pregnancies. Before preserving the uterus, surgeons must offer adequate preoperative counselling and examination (9).

Advantages of Mostafa Maged sling technique include short mean operation time, spinal anesthesia option, avoidance of complications of other techniques in the literature such as ileus, ureter complications and intraabdominal adhesions, injuries to vessels or ureters, etc. By this method, we increase the strength of the sling and avoid losing of stitches of Mostafa Maged technique by suturing the two ligaments together and by taking three knots at different three levels (first level at ligaments, second level at anterior abdominal muscle layer, third level at sheath).

In conclusion, Mostafa Maged sling technique has shown its efficacy on these cases. It is an easy way to treat prolapsed uterus without needing to these alternative methods used in the past which carry a lot of risks such as large vessels injuries, ureter injury, bowel injury, and ischaemia. Thus, we recommend more studies on this Mostafa Maged technique.
